# Integrating disaster risk reduction and climate change adaptation in Seychelles: Challenges and proposed strategies

**DOI:** 10.4102/jamba.v17i2.1808

**Published:** 2025-02-26

**Authors:** Daniel Etongo, Uvicka Bristol, Daniel Cetoupe, Jade Landry, Jean-Claude Labrosse

**Affiliations:** 1 Department of Environmental Sciences, Faculty of Business and Sustainable Development, University of Seychelles, Anse Royale, Seychelles; 2 James Michel Blue Economy Research Institute, Faculty of Business and Sustainable Development, University of Seychelles, Anse Royale, Seychelles; 3 Disaster Risk Management Division (DRMD), Victoria, Seychelles; 4 Department of Energy and Climate Change, Ministry of Agriculture, Climate Change and Environment, Victoria, Seychelles

**Keywords:** disaster risk reduction, mainstreaming, climate change adaptation, vulnerability, resilience, Seychelles

## Abstract

Integrating disaster risk reduction (DRR) and climate change adaptation (CCA) has gained traction among scientists and development practitioners, given their complementary benefits of reducing vulnerabilities and enhancing the resilience of ecosystems, livelihoods and protection of assets while concomitantly enabling the achievements of the Paris Agreement, Sustainable Development Goals and the Sendai Framework on DRR. However, guidance on integrating climate and disaster risk is poorly understood because of insufficient scholarship. Although common concerns exist, the effective integration of DRR and CCA faces diverse challenges in various countries and contexts, and no study has addressed this topic in the Indian Ocean Region and Seychelles. To address this knowledge gap, a content analysis of relevant policies and strategies and in-depth interviews with 40 stakeholders were conducted to identify the challenges of integrating DRR and CCA alongside their proposed strategy for improvement. Barriers that impede the effective mainstreaming of DRR and CCA include issues about (1) governance and politics; (2) policy integration; (3) competing actors and institutions; (4) coordination and collaboration; (5) resources and funding mechanism; (6) scale mismatches; (7) implementation and mainstreaming; (8) community involvement; and (9) information, communication and knowledge sharing. This study reveals that structural coherence was weak on the strategic rather than conceptual levels, leading to poor institutional, operational and financial coordination. This led to incidental integration and collaboration happening on an ad hoc basis with the Disaster Risk Management Division positioned towards emergency preparedness and response. Proposed recommendations to enhance DRR and CCA integration are provided.

**Contribution:**

This study serves as a guide for Seychelles and other countries on how to effectively link DRR and CCA to minimise duplication of efforts and enhance the efficient use of human and financial resources while concomitantly achieving the objectives of DRR – to reduce vulnerability and enhance resilience.

## Introduction

The climate crisis is causing more frequent and intense extreme weather events, with 1.7 billion people affected by hurricanes, cyclones, forest fires, floods and droughts during the last decade (Birkman et al. 2022). Climate-related disasters will become more frequent and intense, putting lives, livelihoods and economic assets at risk (Hallegatte 2014; IPCC 2018; Weerasekara et al. 2021). The United Nations reported that between 1999 and 2018, there was a dramatic increase of 151% in direct economic losses from climate-related disasters globally (United Nations [UN] 2018). The Intergovernmental Panel on Climate Change (IPCC) also projects a further intensification of climate change in the 21st century, including a more frequent occurrence of heat waves, rising wind speeds from tropical cyclones and increasing severity of droughts (IPCC 2023). Just last year, the climate crisis seems to have tightened its grip, with countries in Africa, Europe, Latin America and Southeast Asia hit by climate-induced disasters (Donatti et al. 2024).

In Zambia, the 2023 floods were the worst in the last 50 affecting over 25 000 households and 400 000 people across nine provinces within 2 days of intense rainfall (Ngoma, Finn & Kabisa 2024). Mozambique also suffered from Tropical Cyclone Freddy in 2023, which lasted 35 days and became the most long-lived tropical cyclone in history (Liu et al. 2023). A total of 698 Mozambiquans were injured, 183 died, 123 000 were internally displaced and 132 000 homes were destroyed by flooding that affected over 2 381 354 acres of land and spread waterborne diseases (EM-DAT 2023). Myanmar was also hit by Cyclone Mocha, which caused saltwater intrusion, extensive crop damage and pollution of drinking water sources, affecting 3.4 million of the population. Seven rivers in Ecuador’s northwest Esmeraldas Province overflowed in June 2023 after 12 h of heavy rainfall in June 2023, causing the evacuation of over 500 people (Winckler et al. 2024).

Furthermore, Category 5 Hurricane Otis in October 2023 has been described as one of the strongest storms ever recorded to hit Mexico’s Pacific Coast. According to state reports, Hurricane Otis killed 47 people, destroyed 50 000 homes and damaged an additional 273 844 homes (Garcia-Franco, Gomez-Ramos & Dominguez 2024). Seychelles is not an exception and is vulnerable to various hydrometeorological hazards (Rice et al. 2019). Notable examples include coastal erosion on Praslin in 1986, the massive coral bleaching in 1998, heavy rainfall and coastal flooding on Mahe in 2004, tropical cyclone in 2006 and tidal flooding on Mahe in 2007 and 2012 (Khan & Amelie 2014; Payet & Agricole 2006). In addition, tropical cyclone Bondo in 2004 induced heavy rains and coastal flooding, affecting coastal infrastructures below 2.5 m on Mahe (World Bank & DRDM 2019). Intense rainfall in 2013 overwhelmed the Seychelles’ drainage systems and retaining walls (Government of Seychelles 2013), and flash floods have become frequent during the last two decades (Government of Seychelles 2023).

Furthermore, tropical cyclone Felleng in 2013 caused severe flooding and landslides across the islands of Mahe, Praslin and La Digue, with Cyclone Fantala in 2016 causing substantial damages in the Farquhar Atoll. The total cost of losses and damages to property, agricultural losses and other infrastructures was estimated at 9 million US Dollars (World Bank & DRDM 2019). Two studies in Seychelles reaffirm that hydrometeorological hazards will become more frequent and intense, with heavy rains, flash floods and droughts as typical examples (Cheasty et al. 2017; Government of Seychelles 2023). Such projections imply existing vulnerabilities will be amplified, which can negate developmental gains and economic activities, especially for a country such as Seychelles that depends on climate-sensitive sectors such as tourism, fisheries and agriculture. Therefore, reducing disaster risk and adapting to climate change is not an option but imperative in achieving the 2030 Agenda for Sustainable Development.

In 2015, Members of the United Nations adopted the Sendai Framework for Disaster Risk Reduction (DRR) 2015–2030, the Paris Agreement for Climate Change and the 2030 Agenda for Sustainable Development. The common objectives of these three global agreements are reducing vulnerability and enhancing resilience (United Nations Office for Disaster Risk Reduction [UNDRR] 2020). Recognising the linkages between DRR, climate change adaptation (CCA) and sustainable development, the communities managing the three global processes have started introducing and promoting more coherence in their core agreements. Still, guidance in achieving these noble objectives is scarce. For example, the Sendai Framework reaffirms that DRR is essential for sustainable development, as disasters can derail development plans and reverse development gains. The same framework also recognises climate change as a driver of disaster risk, which, if unmitigated, will enhance the frequency and impacts of disasters (Mizutori 2020; UNDRR 2020). Nonetheless, implementing each agenda has created various institutional arrangements, planning documents, funding mechanisms, and monitoring and evaluation frameworks. There is a need to identify commonalities and differences between these mechanisms to overcome siloed approaches and avoid the duplication of efforts in implementing DRR, CCA and sustainable development with the ultimate objective of fostering risk-informed development (Kelman 2017; UNDRR 2020).

Disaster risk reduction is an approach that strives to reduce disaster risk through systematic and comprehensive efforts that address the causal factors of disasters (Islam, Chu & Smart 2020b). In contrast, CCA approaches strive to adjust to climate change stimuli or their effects, reduce their adverse impacts and exploit beneficial opportunities (IPCC 2014). The standard practice for DRR and CCA is their implementation by different ministries or agencies, as evident in most countries (Dwirahmadi et al. 2023; IPCC 2012; Thomalla et al. 2018), of which Seychelles is not an exception Etongo (2023). The overlap between DRR and CCA has garnered recognition among development practitioners of the need for their mainstreaming, given that development and sustainable goals may be facilitated by such integration. However, guidance on integrating climate and disaster risk is poorly understood because of insufficient scholarship in the Indian Ocean Region (Islam et al. 2020b) and no study has addressed this topic in Seychelles. Although common concerns exist, the effective integration of DRR and CCA faces diverse challenges in various countries and contexts, and no study has addressed this topic in the Indian Ocean Region and Seychelles. This knowledge gap is addressed in the study that seeks to answer the following questions: (1) what are the challenges confronting the effective integration of DRR and CCA in Seychelles? and (2) what are the proposed strategies to address these challenges? The structure of this article consists of the following sections: firstly, a conceptual framework for integrating DRR and CCA; secondly, methodology; thirdly, results; fourthly, discussion and finally, conclusion.

## Conceptual framework – Integrating disaster risk reduction and climate change adaptation

Although some studies have acknowledged the necessity of integrating DRR and CCA (Birkmann & Von Teichman 2010; Dwirahmadi et al. 2023; Islam et al. 2020a; Mercer 2010; Schipper 2009), the mainstreaming process must consider the diverse range of actors and institutions surrounding DRR and CCA. In spite of the fact that these actors and institutions often do not explicitly endorse DRR and CCA integration, the common concern has always been enhancing sustainable development (Islam et al. 2020a, 2020b). Birkmann and Von Teichman (2010) identified the issue of scales, knowledge and norms as challenges confronting the integration of DRR and CCA. Thus, DRR and CCA integration requires combining efforts across the different scales and reducing the mismatches on which the DRR and CCA communities primarily focus (Islam et al. 2020b). Challenges mentioned by other studies regarding the integration of DRR and CCA include a lack of actors’ and institutions’ capacities (Bhatt, Mall & Banerjee 2015; Forino et al. 2017), policy gaps (Islam et al. 2020b; Howes et al. 2014), governance failure (Mall et al. 2019; UNDRR 2020), lack of collaborations and coordination (Begum et al. 2014; Dwirahmadi et al. 2013; Islam et al. 2020b) and funding mechanisms (Islam et al. 2020a; Mall et al. 2019).

According to Djalante and Thomalla (2012), the discussion has passed the stage of justifying ‘why’ the integration of DRR and CCA should occur to the question of ‘how’ the integration should be done. But, achieving the ‘how’ requires guidance that speaks to the realities of different countries and contexts. Studies that have addressed the integration of DRR and CCA have applied different conceptual perspectives. For example, Birkmann and Von Teichman (2010) focussed on (1) spatial and temporal scales, (2) norm systems and (3) knowledge types and sources as essential components for integrating DRR and CCA. Two studies have used collaborative governance as the conceptual framework for enhancing the integration of DRR and CCA (Dwirahmadi et al. 2023; Forino et al. 2017). Although these perspectives are relevant, they fall short of addressing the full spectrum of DRR and CCA integration, given that it is a multistakeholder-driven process that goes beyond governance issues. The UNDRR has developed the most comprehensive conceptual framework, which addresses the full spectrum to effectively integrate DRR and CCA, drawing from a case study across 32 African countries (UNDRR 2020). This framework is adopted in this study.

**FIGURE 1:**
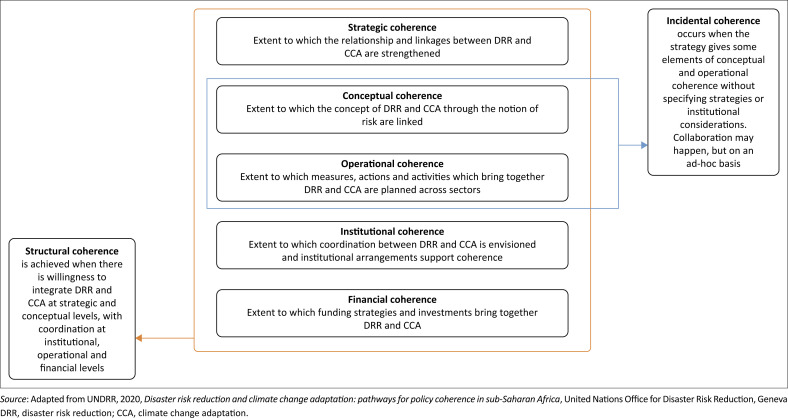
A conceptual framework for integrating disaster risk reduction and climate change adaptation.

The UNDRR assesses the integration of DRR and CCA at three levels – limited, partial or substantial- representing the framework’s strength. Notwithstanding that the study was based on the content analysis of policy documents in the case study countries, the distinction between the different levels of integration was clearly articulated, as presented in Table 1. Moreover, these three levels of integration of DRR and CCA were assessed across five core areas (Figure 1), which are crucial to policies and strategies (UNDRR 2020) and, more importantly, have the potential to address issues beyond governance. Complementing the content analysis with in-depth stakeholder interviews was recommended by the UNDRR to attach meaning to content and help UN Country Teams and member states address country-specific needs, a gap being addressed in this study.

**TABLE 1:** Criteria for assessing disaster risk reduction and climate change adaptation integration in policy and strategic documents.

Themes	Substantial integration	Partial integration	Limited integration
Strategic	Promotes coherence of DRR and CCA, addressing them jointly and mainstreaming DRR and CCA into other sectors	Addresses CCA or DRR as a cross-cutting theme or through one strategic axis. Aiming at mainstreaming DRR into CCA and vice versa	Promotes the integration of DRR or CCA into development and other sectoral policies and plans without specifying which ones
Conceptual	Acknowledges the synergy between DRR and CCA. It considers that CC is a risk factor that increases the frequency and intensity of disaster events. It accounts for CC by including comprehensive climate change projections	It considers that CC is one risk factor and accounts for climate change (cc) by including some projections	Takes into consideration that CC is one risk factor with no climate change projection
Institutional	Mentions a cross-sectoral vertical coordination mechanism to support coherence. Identifies roles and responsibilities linked to DRR and CCA activities with the lead institution mentioned	Mentions the lead CCA agency or the lead DRR agency, but roles and responsibilities are not specific	The lead agency for DRR and CCA is not mentioned
Operational	Identifies common areas of work that cover the DRR cycle (Prevention, Preparedness, Response, and Recovery). Includes specific capacity-building activities for CCA and DRR practitioners	Common work areas are identified but do not cover the entire DRR cycle. More generic capacity-building programmes	Focus on response (emergency services) with no mention of capacity-building programmes
Financial	Promotes the use of DRR funding or CCA funding for both communities. Includes an estimation of the budget in support of DRR and/or CCA activities	Promote the use of DRR and CCA funding separately, with no budget estimate	Promote the use of funding for development projects without specifics on DRR and CCA

*Source*: Adapted from UNDRR, 2020, *Disaster risk reduction and climate change adaptation: Pathways for policy coherence in sub-Saharan Africa*, United Nations Office for Disaster Risk Reduction, Geneva

DRR, disaster risk reduction; CCA, climate change adaptation; CC, climate change.

Strategic coherence examines whether DRR and CCA are explicitly addressed jointly or if the aim is to strengthen the relationship and linkages between the two fields in Seychelles. The Seychelles National Development Strategy is the overarching strategic document guiding national development. This strategy for 2024–2028 is set towards achieving a future that prioritises both growth and resilience for Seychelles (Government of Seychelles 2024). A content analysis of the strategy will reveal whether DRR and CCA are addressed jointly or not.

Conceptual coherence explores how Seychelles links DRR and CCA conceptually, particularly through the concept of risk. A content analysis will be performed on the 10 policy and strategic documents to identify whether the two fields of practice use similar concepts to address issues around DRR and CCA. Conceptual coherence is essential because using similar concepts with the same meaning, irrespective of the field of practice, provides a unified platform for addressing the objectives of DRR – reducing vulnerability and enhancing resilience.

Institutional coherence analyses whether coordination between DRR and CCA is envisioned and whether and how institutional arrangements support coherence. The Ministry of Internal Affairs and the Ministry of Agriculture, Climate Change and Environment (MACCE) are the DRR and CCA leads, respectively. Fostering collaborations and networking with relevant partners is instrumental in harnessing collective efforts and expertise and mobilising resources. To establish strategies for effective DRR-CCA integration in Seychelles, it is essential to explore this integration experience and identify the challenges involved.

Operational coherence looks at measures, actions and activities that bring together DRR and CCA practices and to which extent planning is considered cross-sectoral. The Seychelles National Climate Change Committee (NCCC) was created in 1992, while the National Disaster Committee (NDC) was created in 1995 (DECC 2022; DRDM 2014). While the latter’s mandate was to study the different natural hazards that may affect the country, the former guided the nation on climate change and its impact. The mandates of both committees are complementary. However, they are coordinated by different ministries, and cross-sectoral integration of DRR and CCA actions and activities might be constrained.

Financial coherence explores whether and how funding strategies and investments bring together DRR and CCA. Financial flow for DRR and CCA-related projects and programmes in Seychelles, including sources and purpose from relevant documents and, more importantly, key informant interviews, is essential. This approach provides an overview of the funding landscape for DRR and CCA interventions in Seychelles by identifying overlaps, gaps and areas in which DRR and CCA finances have reinforced each other.

Moving to a more substantial integration of DRR and CCA in policy documents means advancing towards a structural approach to coherence whereby strategic, conceptual, institutional, operational and financial coherence is achieved (UNDRR 2020). This would require particular emphasis on conceptual and institutional coherence to clarify roles and responsibilities and set a basis for coherent cross-sectoral implementation of DRR and CCA. Institutional arrangements are a cornerstone of structural coherence and should be strengthened (UNDRR 2020). The critical question becomes how can progress for this integration be achieved? For example, a study in Mexico by Ruiz-Rivera and Lucatello (2017) addresses the interplay of actors and institutions surrounding DRR and CCA and their influence on integration (Islam et al. 2020b).

In contrast, De Leon and Pittock (2016) documented the DRR and CCA policy integration progress in the Philippines. A paucity of literature exists on the small island developing states (SIDS) of the Indian Ocean, and the only study from Mauritius focussed on learning from risk reduction pilot projects by demonstrating the integration of DRR and CCA (Anisimov, Magnan & Duvat 2020). A study from Bangladesh reviewed several studies on DRR and CCA integration challenges, but it was not anchored on a specific framework. The UNDRR offers the most recent and comprehensive framework on this topic. More local-level case studies are required to assess and evaluate the potential strategies for successfully integrating DRR and CCA in different contexts. Against this backdrop, to what extent has Seychelles made progress along this path, and what challenges DRR-CCA practitioners are still facing are yet to be explored and constitute the focus of this study (Islam et al. 2020b).

## Research methods and design

### An overview of disaster management legislation in Seychelles

The Disaster Risk Management (DRM) Policy of the Republic of Seychelles and the Disaster Management Act of 2014 set the structure and guidance for DRR and response in the Seychelles. These documents highlight the government’s responsibility for protecting communities and the environment by establishing the Disaster Risk Management Division (DRMD) as the focal point and primary national body for DRR and emergency management. The statutory responsibilities of the DRMD cut across 11 key areas, some of which include (World Bank & DRDM 2019): (1) Identify and prioritise risks to ensure that existing services are prepared and equipped to deal with realistic potential emergencies; (2) Ensure preparedness by the principal response agencies to ensure prompt and effective coordinated response; (3) Ensure that measures are in place to restore livelihoods and other life support; (4) Coordinate response in the event of a threat of disaster; and (5) Advise, assist and coordinate the activities of government institutions, non-governmental organisations, private sector entities and communities, among others (UNDRR 2020).

**FIGURE 2:**
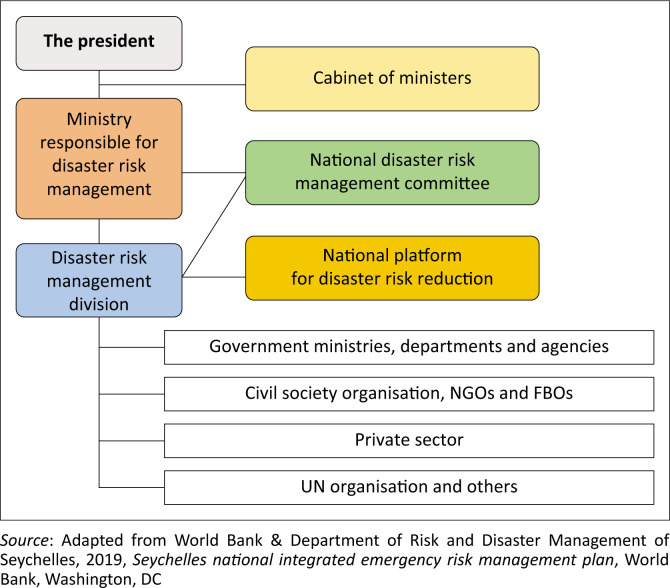
Governance structure of disaster risk management in Seychelles.

The DRDM Act 2014 provides the establishment of two main bodies, the National DRM Committee and the Vulnerability Assessment Committee, along with the establishment of a platform, the National Platform for DRR (DRDM 2014; UNDRR 2020). The committees and platform comprise members across relevant Ministries, Departments and Agencies (MDAs). Disaster risk reduction is aimed at preventing new and reducing existing disaster risk and managing residual risk, all of which contribute to strengthening resilience and, therefore, to achieving sustainable development. This explains why DRR and management in Seychelles is a whole-country, multistakeholder approach, including state and non-state actors, international Non-Governmental Organizations (NGOs) and donor organisations (Figure 2). Going by the governance framework, the Ministry of Internal Affairs reports directly to the president, and disaster management issues are brought to the cabinet of ministers. The DRMD, the implementing entity, works alongside other MDAs, including several non-state actors, as presented in Figure 2. Therefore, DRM is cross-cutting across all MDAs and the DRMD coordinates these activities as outlined in its statutory responsibility (World Bank & DRDM 2019).

### Data collection

The entry point, which is the first stage of this study was the selection of policies, strategies and action plans in Seychelles that address issues of DRR and CCA. After careful screening, 10 documents that address this issue were retained (Table 2). These documents include climate change, DRM, land use plans, coastal management and blue economy development. These documents were reviewed based on the assessment criteria in Table 1, which addresses the level of DRR and CCA integration across five thematic areas.

**TABLE 2:** Reviewed documents relevant to disaster risk reduction and climate change adaptation.

Document title	Source and year
Seychelles National Integrated Emergency Management Plan	(World Bank and MEECC 2019)
Seychelles Sendai Framework for Disaster Risk Reduction – Mid-Term Review Report	(DRMD 2022)
Seychelles National Disaster Risk Reduction Strategic Plan 2021–2030	(DRMD 2021)
Seychelles Coastal Management Plan 2019–2024	(World Bank and MEECC 2019)
Seychelles Strategic Land Use and Development Plan	(GOS 2015)
Seychelles National Climate Change Policy	(GOS 2020)
Seychelles Updated Nationally Determined Contribution to the UNFCCC	(GOS 2021)
Seychelles Third National Communication to the UNFCCC	(GOS 2023)
Seychelles Blue Economy: Strategic Policy Framework and Roadmap – Charting the Future 2018–2030	(GOS 2018)
Seychelles National Development Strategy 2024–2028	(GOS 2024)

**FIGURE 3:**
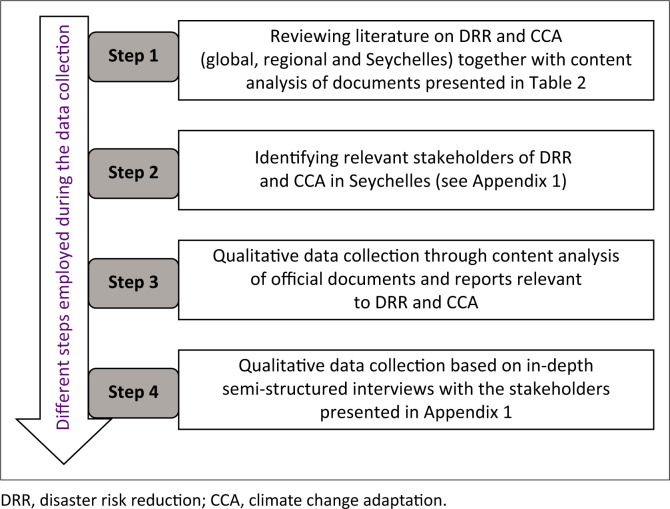
Data collection methodology applied in this study.

The second stage of the data collection process was identifying relevant stakeholders on DRR and CCA alongside conducting an in-depth semi-structured interview, as presented in Figure 3. These stakeholders are those involved in the design of policies and strategies including the implementation of DRR and CCA-related programmes and projects in Seychelles. They include policymakers and technicians from six government MDAs, from the academia, private sector, and local and international NGOs (see Appendix 1 for details). Using a qualitative semi-structured interview approach allows for the identification of the meanings people attribute to their experiences – in this case, regarding the integration of DRR and CCA. The semi-structured interviews explore the perspectives of the practitioners on the extent to which CCA is integrated into their practice of DRR in Seychelles as follows: (1) understanding of CCA; (2) linkages between CCA and DRR; (3) links between CCA and land use planning; and (4) obstacles, gaps and opportunities for integrating CCA with DRR and national development. The interviewees were selected from the national DRR strategic plan (DRMD 2021), which contains a comprehensive list of all the Seychelles stakeholders involved in DRM. The aim of the research was explained to all the respondents, and their consent was sought verbally before the interview, which lasted from 45 min to an hour. A total of 40 semi-structured interviews were conducted. For a complete list of interviewed stakeholders, see Appendix 1.

### Data analysis

The complementary qualitative data from strategy and policy documents and stakeholder interviews were analysed using the MAXQDA 2018 version. The PDF versions of strategy and policy documents considered in this study were uploaded in the MAXQDA interphase. This qualitative data analysis software program is designed for computer-assisted qualitative and mixed methods. This software was used because of its ability to find themes or relationships in documents or other text sources. It involved four main steps (Adam, Pretzsch & Darr 2015) that were adopted in this study as follows: (1) identification of the main themes; (2) attributing codes to the main themes; (3) classification of responses under the main themes; and (4) integration of themes and responses into narratives (Etongo 2019).

### Ethical considerations

Ethical clearance to conduct this study was obtained from the University of Seychelles Research Ethics Review Committee received on 30th August 2024.

## Results

Findings from the in-depth content analysis of relevant documents and stakeholder interviews on DRR and CCA integration are presented in three sections. The first part of the results focusses on the progress made towards implementing the Sendai Framework. This is followed by the challenges faced in linking DRR and CCA and the proposed strategies to enhance the integration of DRR and CCA in Seychelles.

### Progress in implementing the Sendai framework

At the time of the Mid-Term Review of the Sendai Framework, Seychelles has made some progress towards achieving some of the targets set out by the framework. Notable achievements include establishing focal persons to amass data and report on incidents or events, along with developing a National DRR Strategic Plan (2021–2030). Another milestone is the establishment of a National Platform for DRR. In February 2022, 13 officials from various MDAs were appointed as members of the Seychelles Vulnerability Assessment Committee (Sez Vac) for a 3-year term by the Minister for Internal Affairs, who is responsible for the DRM portfolio nationally. The committee, which the director general of the DRMD chairs, has the following responsibilities: (1) to make specific recommendations on the processes and development strategies to reduce vulnerabilities; (2) to provide the government with relevant information on poverty reduction strategies and safety net programming relevant to DRR; and (3) to identify various hazards and assess risks that could give rise to food and nutrition vulnerability nationally.

Despite such progress towards the implementation of the Sendai Framework in Seychelles, the nation still faces crucial challenges across the four priorities of action as follows (UN 2015): ***Priority 1: Understanding disaster risk*** – (1) the vulnerability in Seychelles is multi-dimensional, and the country still faces significant data gaps, particularly in the outer islands; and (2) Seychelles requires adequate technology and capacity to strengthen its risk analytics and disaster loss data collection; ***Priority 2: Strengthening disaster risk governance*** – (1) Seychelles have been pioneering the integration of DRR and CCA into unified plans through the creation of the NDC in 1995. However, implementation has been slow because of the siloed approach to project development, funding streams and governance arrangements; and (2) Seychelles is unable to tackle the full spectrum of hazards it faces because of the lack of commitments from some MDAs, and limited local capacity; ***Priority 3: Investing in disaster risk reduction*** – (1) The amount of financing does not match the scale of existing and emerging disaster risks in Seychelles; (2) the composition of available financing is highly skewed towards response, with few resources being dedicated to pre-emptive resilience building; and (3) risk considerations are not yet integrated into development and financial decisions, which leads to maladaptation and the creation of new risks; ***Priority 4: Enhancing preparedness for response and to build back better*** – (1) Seychelles still lags in multihazard early warning coverage; and (2) the challenges with access to finance mean that Seychelles stays in perpetual recovery phases and miss opportunities to build back better.

### Issues and challenges confronting disaster risk reduction and climate change adaptation integration

Findings from the literature (Islam et al. 2020b; UNDRR 2020), relevant DRR and CCA documents in Seychelles (see Table 2), and the in-depth interviews with the 40 stakeholders about the challenges of integrating DRR and CCA are grouped into nine themes as follows: (1) governance and politics; (2) policy integration; (3) competing actors and institutions; (4) coordination and collaboration; (5) resources and funding mechanism; (6) scale mismatches; (7) implementation and mainstreaming; (8) community involvement; and (9) information, communication and knowledge sharing. These challenges are discussed in light of the literature and quotes from in-depth interviews that reflect the Seychelles context.

#### Governance and politics

The governance framework for DRM in Seychelles highlights the onus to invest in DRM lies with the state (World Bank & DRDM 2019). In contrast, preventing and reducing risk remains a shared responsibility among MDAs, which must act in the state’s interest. Despite being a shared responsibility, the DRMD within the Ministry of Internal Affairs must coordinate across MDAs and other non-state actors to ensure risk reduction, disaster management and recovery are achieved. The DRM Act (2014) makes provisions for establishing two main bodies, the National DRM Committee and the Vulnerability Assessment Committee, created in 1995 and 2022, respectively. While the DRMD chairs both committees, a NCCC was created in 1992 and chaired by the Department of Energy and Climate Change in the MACCE. These committees have existed for nearly three decades, and the integration of DRR and CCA is still in its infancy.

One of the respondents (R006) mentioned that:

‘[*S*]ome of the recommendations in the coastal management plan have been ignored, and development projects have been implemented in areas where new risks will emerge. Most of our development has occurred along the coastline, where most hazards occur. It will occur in the future, and disaster risk reduction, which is much cheaper than adaptation, has not been given priority.’ (41 years, male, DRMD)

Another respondent (R0014) opined that:

‘[*W*]e cannot separate most of our development projects from DRR and CCA even if different MDAs implement them, and that is why we have a national development strategy. Development projects should help us reduce climate-related risk and enhance our adaptive capacity rather than expose us to more vulnerability.’ (45 years, female, local environmental consultant)

Disaster risk reduction, CCA and development projects in the case of Seychelles often involve, in most cases, infrastructural projects that are implemented by four key ministries – MACCE, Ministry of Fisheries and Blue Economy, Ministry of Transport, and the Ministry of Lands and Housing. Because every DRR and CCA project occurs within a district, the Ministry of Local Government and Community Affairs, through its various District Administrators (DAs), is part of the advisory team. In principle, the governance framework for DRM in Seychelles involves a whole-country approach that includes state and non-state actors. However, a grey area regarding the monitoring and evaluation of DRR persists. For example, all MDAs are supposed to submit periodic reports on their activities and projects that link to DRR and management in the country to DRMD. Information gathered during the data collection showed that although some MDAs have appointed contact persons, the required information is often not provided.

#### Policy integration

A comprehensive content analysis of the 10 policy and strategic documents (see Table 2) revealed that DRR and CCA have been integrated across sectors. The Seychelles Updated Nationally Determined Contribution highlights this integration, with adaptation being a national priority, of which DRR is a core component. The policy vision of the national climate change policy of ‘a sustainable, climate-resilient and low-carbon Seychelles’ also reaffirms the integration of DRR and CCA. Priority area six of Seychelles national development strategy 2024–2028 focusses on environmental sustainability and climate change resilience with integrating DRR and CCA. Some interviewees corroborated this view that integrating CCA into DRR regulation and DRR into CCA regulation has made significant progress in national policies, strategies and action plans. However, translating policies and strategies into actual implementation seems to be the main challenge.

One of the participants (R001) responded:

‘Seychelles has yet to develop a National Adaptation Plan (NAP) due to a delay of over two years in receiving funding from the Global Environment Facility. The NAP process seeks to identify medium- and long-term adaptation needs, as informed by the latest climate science, now available in our Third National Communication to the UNFCCC. Once major vulnerabilities to climate change have been identified, the NAP process develops strategies to address them, which is the best way of integrating CCA into DRR while concomitantly achieving the vision of the climate change policy and priority 6 of the national development strategy – environmental sustainability and climate change resilience.’ (36 years, female, MACCE)

Another respondent (R005) mentioned that:

‘[*M*]ajor policies in Seychelles such as the climate change policy, nationally determined contribution, national integrated emergency management plan, and national development strategy are clear in addressing DRR and CCA. This is because the IPCC, UNDRR, UNFCCC, and SDG guide them. However, some guidelines need to be implemented to enhance the integration of DRR and CCA into the operations of relevant MDAs in Seychelles.’ (48 years, male, Seychelles Meteorological Authority [SMA])

#### Competing actors and institutions

The Ministry of Internal Affairs leads DRR-related planning and efforts through the DRMD. In contrast, the MACCE leads CCA-related planning and efforts through the Department of Energy and Climate Change in Seychelles. Other ministries also influence the planning and implementation of DRR and CCA-related activities, such as the Ministry of Lands and Housing, the Ministry of Transport, the Ministry of Finance, Economic Planning and Trade, and the Ministry of Local Government and Community Affairs. A total of 90% of respondents stated that competing and conflicting interests are evident among some government MDAs in controlling decisions regarding integrating DRR and CCA. For example, most DRR projects are implemented by the MACCE rather than the DRMD, with minimal input from the latter. The need for clarity of mandate among institutions implementing DRR-related projects is needed to avoid conflicts and overlap.

#### Coordination and collaboration

The DRMD coordinates DRM through the National Committee on Risk Reduction. On the other hand, CCA falls under the responsibility of the Department of Climate Change in the MACCE. The DRMD and the MACCE have some form of collaboration, especially in projects and the development of national documents. For example, the development of the Seychelles Coastal Management Plan 2019–2024 witnessed the active participation of the DRMD. The Department of Climate Change at the MACCE has mandated one of its Principal CCA Specialists as the contact person to the DRMD to enhance their working relationship. Other MDAs’ collaboration has been ad hoc, especially during specific projects, as was the case during the development of the Seychelles National Integrated Emergency Management Plan. The National Committee on Risk Reduction, which the DRMD coordinates within the Ministry of Internal Affairs, plays a vital role in national risk reduction management matters. However, this committee has witnessed poor participation of MDAs, partly because of a lack of understanding of risk reduction management and its importance nationally, as well as a lack of synergy among MDAs and, by extension, the National Development Strategy.

#### Resources and funding mechanism

**FIGURE 4:**
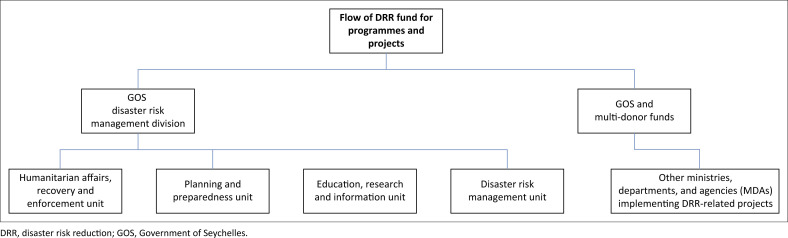
Flow of funding for the implementation of disaster risk reduction programmes or projects in Seychelles.

Regarding financial resources, the DRMD largely depends on the national budget as part of its allocation within the Ministry of Internal Affairs for the implementation of projects and programmes (Figure 4). The implication is that several projects that have been designed across the four operational units of the DRMD are yet to be actualised. Human and financial resources have been received ad hoc, such as during the development of the Seychelles National Integrated Emergency Management Plan. On the other hand, CCA has received considerable attention regarding financial and human resources from national and international institutions and organisations. For example, country allocation for Global Environment Facility (GEF) funded projects are often directed to CCA. Designated funds such as the Special Climate Change Fund (SCCF) exist without dedicated funding for DRR. Funding is also provided by the GEF, UNDP and other international organisations for project implementation. However, some of these projects directly benefit DRR, given that the Department of Climate Change at the MACCE has the following responsibilities: coastal protection and management, coastal erosion and control, and wetland management.

The DRMD continues to face the challenges of accessing funding from international organisations. Disaster risk reduction and CCA funds are managed from divergent sources and the funding distribution process is complex and multilayered. Moreover, although Seychelles is not a carbon-emitting country, it is vulnerable to the impacts of climate change, with approximately 90% of its critical infrastructure, such as schools, roads, airports, hospitals, water and power lines, located along the coast. Financial resources are urgently needed for DRMD to develop and promote community-based early warning systems (EWSs), among other projects such as place-based vulnerability assessment.

#### Scale mismatches

Scale mismatches in DRR and CCA relate to spatial, temporal and functional scales. Climate change issues are often analysed nationally, whereas disasters focus on localities where they occur. While climate projections in Seychelles are based on regional models downscaled to the national level, the DRR community focusses primarily on local vulnerabilities and risks of specific areas, hazards and groups of people potentially or affected. The lack of local, down-scaled data on climate change effects or the localisation of the impacts of extreme events in the future (e.g. heavy rainfall, floods and saltwater intrusion) that could facilitate the preparation of specific adaptation and DRR strategies is one of the major concerns of risk reduction and adaptation managers in Seychelles (Birkmann & Von Teichman 2010).

Disaster risk reduction, in general, aims to support sustainable development. Consequently, DRR measures are intended to be long-term oriented. However, this is not always the case in Seychelles and elsewhere, given that humanitarian assistance from the United Nations, various aid agencies and national donor programmes in Seychelles for disaster response are often event-related and, therefore, short-term in its interventions and procedures. Instead of encouraging comprehensive vulnerability reduction and adaptation measures through process-oriented funding, these schemes focus mostly on emergency response, with little attention given to another aspect of the disaster management cycle. Additionally, the negative effects of adaptation measures are not sufficiently considered because they can be detected solely on a later temporal scale. For example, the predominant use of air-conditioning in Seychelles to adapt to higher temperatures contributes to global warming in the future because over 90% of the energy consumed is from fossil fuels (Birkmann & Von Teichman 2010).

Functional scale mismatches refer to the organisation and management of crises and CCA by actors affiliated with different institutions and the distribution of responsibilities, which are often challenging. In Seychelles, the MACCE tackles climate change issues that involve the implementation of projects and programmes to enhance the resilience of ecosystems and the environment to the impacts of climate change. In contrast, DRM lies within the responsibility of the Ministry of Internal Affairs. Differences in their respective mandates, programmes and sets of measures on how to deal with climate change issues, on the one hand, and DRR, on the other hand, create great difficulties when developing a coherent and integrative strategy (Birkmann & Von Teichman 2010).

#### Implementation and mainstreaming

The cross-cutting nature of DRR and CCA in Seychelles and elsewhere implies multiple sectors and actors within a multilevel governance structure with different power relations and resource access are part of the process. Disaster risk reduction and CCA were integrated into relevant policies and strategies (climate change policy, NDC, national disaster reduction strategic plan, coastal management plan and national development plan). Still, their implementation and mainstreaming proved difficult because of sector-based rather than cross-sectoral approaches. Therefore, mainstreaming DRR and CCA into sector policies and strategies is still poorly understood, given that it has occurred ad hoc through different projects rather than being a process that addresses sectoral policies as part of the whole.

One of the respondents (R002) pointed out that:

‘[*T*]he move by the Government of Seychelles to protect 50% of its blue carbon ecosystems by 2025 and 100% by 2030 is a step in the right direction. However, a recent decision by the government to embark on a multi-phased land reclamation project at the Providence Industrial Area on Mahe Island by the end of the year raises a question of the initial commitment by the government.’ (46 years, male, academia)

On the other hand, another respondent (R0015) mentioned that some success stories have been achieved in DRR and CCA implementation and mainstreaming in Seychelles. In this regard, the ecosystem-based adaptation to climate change Seychelles project was cited as:

‘[*F*]reshwater wetland ecosystems were rehabilitated through this project. The rehabilitation led to the return of water used by the farming community to irrigate their crops rather than depending on the water from the Public Utilities Corporation (PUC), 80% of which comes from desalinisation. Also, native biodiversity has been restored at these sites, and the water is available all year round given that a locally designed retaining wall was constructed to regulate the flow capacity in the wetland. This project is a classic example of the water-energy-food (WEF) nexus and has gained traction in Seychelles with other degraded freshwater wetlands being rehabilitated by the Department of Agriculture.’ (48 years, female, UNDP)

The involvement of local communities and farmers in this project emphasises that public–private partnerships are crucial for resource-use efficiency and sustainability of DRR and CCA implementation and mainstreaming.

#### Community involvement

The DAs within the Ministry of Local Government and Community Affairs are the entry point towards community involvement in DRR efforts. Communities have long experienced disaster and environmental impacts, as evidenced by several interventions based on Local Ecological Knowledge (LEK) that have been applied to reduce coastal erosion, landslide and water scarcity. However, the active engagement of communities in the planning, implementation, monitoring and evaluation of DRR and CCA-related projects is an area of concern that requires significant improvement. Eighty-five per cent of interviewees claimed that the community comprises the ultimate beneficiaries who deal with disaster and climate risks. It was evident that community involvement in DRR and CCA-related projects has improved during the last decade as project identification, especially for GEF-funded projects, often takes a whole-country approach with the active participation of community members. Therefore, aside from GEF-funded projects, other DRR and CCA-related projects should be consulted with community members before planning, designing, selecting locations and implementing them.

One of the respondents (R009) mentioned that:

‘[*C*]ommunity members were involved in the ecosystem-based adaptation project that led to the creation of five watershed committees. These community members benefited from different pieces of training that enabled them to manage these watersheds after the project was completed. Baie Lazare is the most active of these five watershed committees and has developed a watershed management plan for 2022–2026. Most of the committee members in the Baie Lazare watershed committee are farmers who also benefit from the water within the upland wetlands for irrigating their farmlands. Therefore, community involvement in DRR and CCA-related projects requires insights on those with a stake to ensure active rather than passive engagement.’ (46 years, male, local environmental consultant)

#### Information, communication and knowledge sharing

Seychelles has vast and varied experience in CCA, given that adaptation is considered a national priority that has attracted more financing from national and international institutions. In contrast, DRR has not received the same attention and has little experience dealing with climate-induced disasters. Therefore, CCA practitioners should share their experiences with DRR practitioners. Some pilot projects on environmental impact assessment have been implemented. However, respondents have agreed that Seychelles still does not have any countrywide disaster and climate change-related vulnerability assessment for each district and subdistrict (Islam et al. 2020b). Such a national study will provide baseline information on vulnerability hotspots in Seychelles, which will, in turn, guide proactive interventions with the potential of reducing the occurrence of climate and anthropogenic-induced disasters.

In February 2022, 13 officials from various public sector organisations were appointed as members of the newly established Seychelles Vulnerability Assessment Committee (Sez Vac) for a 3-year term by the Minister for Internal Affairs, who is responsible for the DRM portfolio nationally. Section 14 of the DRM Act 2014, the law that governs the functions and operations of the DRMD, makes provision for establishing the committee. The director general of the DRMD chairs the committee and has as its general functions the following: (1) the task to provide specific recommendations on the processes and development strategies to reduce vulnerabilities; (2) inform the government of poverty reduction strategies and safety net programming relevant to DRR; and (3) identifying various hazards and assessing risks that could give rise to food and nutrition vulnerability nationally. While this is a great initiative, communication and knowledge sharing from the committee have not been feasible in the public space.

### Proposed strategies to enhance disaster risk reduction and climate change adaptation integration

Despite the numerous challenges highlighted by interviewees towards integrating DRR and CCA, proposed strategies to enhance the mainstreaming process that could reduce vulnerability and enhance Seychelles’ adaptive capacity were provided. These proposals address all the five core areas of the conceptual framework for this study – strategic, conceptual, operation, institutional and financial integration of DRR and CCA. The proposals for improvement from the relevant stakeholders are presented across the four priority areas of the Sendai Framework for DRR. In addition to using risk assessments to inform coherent policymaking, knowledge of risk should be enhanced at all levels of society through formal and informal education, public awareness and citizen participation (UNDRR 2020).

#### Priority 1: Understanding disaster risk

In Seychelles, there is a need to invest and dedicate resources to data collection for inner and outer islands and to develop risk assessments that can inform policymaking. Given the commonality of issues addressed by the DRMD and the Department of Energy and Climate Change (DECC) in Seychelles, the following actions were proposed to enhance the integration of DRR and CCA:

- The DRMD and the DECC should develop or agree on common methodologies for hazard and vulnerability assessments. This would help to focus on all projects similarly and allow relevant MDAs to repeat successful aspects and learn from mistakes, resulting in a continuous improvement process. A common methodology is a great tool for generating efficiency as it is used
- A comprehensive risk assessment for the entire Seychelles Islands should be developed and the results should be shared to enhance knowledge, discussion and feedback
- The capacities of DRR and CCA policymakers and experts should be strenghthened on using risk assessments for risk-informed policymaking, such as strategies and action plans for DRR and CCA, sectoral policies and national development strategies. The Ministry of Finance, National Planning, and Trade leads the development of the Seychelles National Development Strategy through a multistakeholder approach. Yet, some of its senior management staff do not fully understand issues around DRR and encourage certain development projects that increase vulnerability
- Map data should be available for hazard and vulnerability assessments to identify gaps and enhance data availability, sharing and repositories (UNDRR 2020)
- Collaboration between the DRMD, DECC, SMA, National Bureau of Statistics Seychelles, all MDAs, the University of Seychelles and other non-state actors should be promoted to develop comprehensive risk assessments.

#### Priority 2: Strengthening disaster risk governance to manage disaster risk

The difference in institutional arrangements between DRR and CCA has created challenges for policy coherence regarding data and information exchange, planning processes, funding schemes, and monitoring and reporting. Breaking silos between DRR and CCA requires coherent governance systems, which build upon legal frameworks, policies, coordination mechanisms, strong leadership, clear roles and responsibilities, resources, monitoring, and accountability set up across sectors and at all levels. Cross-sectoral and multi-sectoral approaches are indeed key to building a common understanding (UNDRR 2020). The proposed strategies to enhance the integration of DRR and CCA include the following:

- Strengthen the National Bureau of Statistics to produce and centralise data and information related to risk assessments and indicators
- Capitalise on monitoring and reporting processes, such as Seychelles’ voluntary national report for the midterm review of implementing the Sendai Framework for DRR 2015–2030 to enhance coherence among DRR and CCA
- Ensure the systematic integration of DRR and CCA policymakers and experts in existing inter-ministerial and multistakeholder platforms and committees, such as the DRR Platforms, NDC and climate change committee
- Use the national development strategy involving DRR and CCA lead implementing agencies and experts as an entry point to ensure structural integration through strategic, conceptual, institutional, operational and financial coherence
- Convene multistakeholder peer-learning exchange to review information, identify opportunities to harmonise policy, strategies, monitoring and evaluation frameworks, and address capacity gaps (UNDRR 2020)
- Increase awareness and understanding of the coherence of MDAs by disseminating advocacy tools and facilitating training and peer-learning exchanges.

#### Priority 3: Investing in disaster risk reduction for resilience

In coherence with national development plans, DRR strategies should provide the path to increase investment for resilience by involving state and non-state actors. However, Seychelles shows limited investment in DRR and risk financing – a common problem in developing countries face when integrating financial considerations into DRR strategies. Proposed strategies from the respondents include:

- Conduct risk-sensitive budget and expenditure reviews and use them to advocate for investments in and budget support for DRR and CCA
- Enhance awareness of the role of the private sector for DRR and CCA and advocate for its involvement to enhance resilience coherently
- Facilitate dialogue among the Seychelles Ministry of Finance, National Planning and Trade, DRMD, and DECC to identify priority interventions, convene actions for increased resilience investment, and optimise sources of funding and domestic resources
- Convene DRR and CCA actors together with the Ministry of Finance, National Planning, and Trade to define methodologies and approaches for tracking investments and expenditures in DRR and CCA, respectively
- Promote collaboration between DRR and CCA actors to support the development of risk financing strategies combining budget support, risk transfer and insurance mechanisms (UNDRR 2020).

#### Priority 4: Enhancing disaster preparedness for effective response and building back better

Disaster preparedness to ‘Build Back Better’ in recovery, rehabilitation and reconstruction is a key area for integrating CCA and DRR, and it should be further harnessed. The Sendai Framework calls for developing capacities that reduce disaster during recovery. Climate change impact scenarios must be part of risk assessments and informed planning guiding recovery, rehabilitation and reconstruction. They are instrumental in ensuring that new infrastructures are resilient to future climate changes and arbitrating long-term decisions (UNDRR 2020). Proposed strategies from stakeholders are as follows:

- Ensure systematic integration of adaptation to inform recovery planning through capacity-building, comprehensive risk assessments and coordination mechanisms
- Strengthen coordination mechanisms to define implementation modalities in areas of common concern for DRR and CCA strategies, especially those dealing with preparedness, EWS and emergency response
- Coordinate the coherent application of social protection, insurance and risk transfer mechanisms foreseen by DRR and CCA strategies for the response and recovery phases
- Promote policy dialogue among the SMA, CCA and DRR stakeholders to clarify roles and responsibilities in EWS and optimise interventions in capacity-building, data availability, standard operating procedures and linkages to response and adaptation (UNDRR 2020).

## Discussion

The results from the content analysis of relevant policies, strategies and action plans complemented by 40 stakeholder interviews have clearly outlined some common concerns of the challenges confronting the integration of DRR and CCA in Seychelles. The information gathered during the interviews shows that integrating DRR and CCA in Seychelles has made substantial and partial progress in policies and some practices. However, a lack of integration still exists in many sectors, policies, programmes and project implementation in the inner and outer islands. The key challenges in the integration of DRR and CCA in Seychelles include: (1) governance and politics; (2) policy integration; (3) competing actors and institutions; (4) coordination and collaboration; (5) resources and funding mechanism; (6) scale mismatches; (7) implementation and mainstreaming; (8) community involvement; and (9) information, communication and knowledge sharing.

Interestingly, our study found that integrating DRR and CCA occurs more often on an ad hoc basis, implying more incidental than structural coherence, as supported by previous studies from South Asia and sub-Saharan Africa (Islam et al. 2020a, 2020b; Mall et al. 2019; UNDRR 2020). Although conceptual elements show that there is recognition that DRR is linked to climate change and operational elements indicate overlapping activities, there is rarely an indication that these are the results of a collaborative process (UNDRR 2020). The Seychelles NCCC and the NDC were created in 1992 and 1995, respectively (DECC 2022; DRDM 2014). These committees have existed for nearly three decades and led CCA and DRR-related activities in the country but have yet to integrate DRR and CCA structurally.

Seychelles has a strong legal and policy background regarding DRR and CCA. Disaster risk reduction-related policies emphasised CCA and CCA-related policies emphasised DRR. Nevertheless, the key issue is fragmentation in their implementation by separate departments and ministries – a view supported by previous studies from Bangladesh and the Philippines (De Leon & Pittock 2016; Islam et al. 2020b; Rahman et al. 2019). Therefore, both approaches need to have integrated implementation processes at all levels. According to the UNDRR (2020), structural coherence is achieved when there is a willingness to integrate DRR and CCA at strategic and conceptual levels, with coordination at institutional, operational and financial levels. In the case of Seychelles, more integration occurred at the conceptual level than at the strategic level.

For example, hazards frequently found in DRR and CCA strategies are floods, droughts, saltwater intrusion, tropical cyclones, storms, landslides, landfills and forest fires. This implies that both strategies envision actions to prepare and adapt to the impacts of these hazards –frequently called ‘extreme events’ by the CCA practitioners. However, in the absence of details on institutional coherence, it is unclear to what extent the consideration of these areas in both strategies is the result of dialogue and coordination or represents rather a convergence in the understanding that both communities have of their areas of work, responsibilities and contributions to enhance resilience. The UNDRR reiterated that institutional coherence is the key to achieving policy coherence between the DRR and CCA (UNDRR 2020).

The DECC has recently appointed a contact person to work closely with the DRMD. While this initiative is applauded, there is a need to strengthen DRR and CCA governance in policies, outlining how responsibilities will be attributed and how coordination will happen. Governance challenges and inadequate coordination and collaboration have been identified as issues compromising the effective integration of DRR and CCA in Bangladesh, Australia and Indonesia (Dwirahmadi et al. 2013; Gaillard et al. 2013; Howes et al. 2014; Islam et al. 2020b). Strengthening institutional coherence will allow us to move from incidental coherence, the common outcome in Seychelles, to structural coherence. Institutional coherence could then contribute to avoiding duplication of efforts and resources by providing a platform for discussing the allocation and prioritisation of funds and activities based on the comparative advantage and expertise of both practices (UNDRR 2020). In this context, the Seychelles National Development Strategy is a useful tool that clarifies objectives, responsibilities and budget and, as such, can support institutional, operational and financial coherence (Government of Seychelles 2024; UNDRR 2020).

Regarding financial resources for DRR and CCA, more funding was allocated to the latter than the former, with DRMD largely dependent on government funding. Such skewness in the financial landscape is expected, given that the statutory mandate of the DRMD is more about coordinating all the MDAs towards implementing DRR projects and programmes. For example, most DRR projects are implemented by the MACCE, attracting more finances from external donors. Inadequate financial resources were considered a crucial barrier that limited several aspects of integrating DRR and CCA. It is important to reiterate that financial resources will never be sufficient, and alternative pathways to optimise the usage of available resources are vital. For example, developing risk financing strategies combining budget support, risk transfer and insurance mechanisms to advanced DRR and CCA planning will reduce the duplication of efforts and increase the efficient use of existing funds, a strategy supported by Pervin et al. (2019).

Another issue about finance in national plans and strategies, except for the Updated Nationally Determined Contribution (Government of Seychelles 2021), is the lack of cost of planned activities, so implementation is compromised. Nonetheless, considering hazards such as floods and droughts in the domain EWS implies that climate finance mobilised for implementing CCA strategies such as GEF, Green Climate Fund (GCF) and the Adaptation Fund is being allocated and leveraged for actions related to DRR. The ecosystem-based adaptation to climate change Seychelles project that the Adapted Fund funded is a good example. Degraded freshwater wetlands were rehabilitated through this project to solve the drought problem among farming communities that use the water to irrigate their crops (Etongo, Vel & Mendez 2022). This provides an entry point for financial coherence, which should be explored further.

The DRMD is mandated to coordinate DRR in the country through a multistakeholder approach of MDAs and other non-state actors. The NDC was created to deliver on its mandate, with participants from all the relevant stakeholders involved in DRR and CCA-related activities. The Climate Change Committee has existed since 1992 and has participants from MDAs, including the private sector, local NGOs and academia (DECC 2022). Despite having these fora that promote the engagement of DRR and CCA experts, minutes are hardly taken during these meetings. This makes it difficult to grasp to which extent DRR and CCA experts directly engage, notably regarding information exchange and implementation (UNDRR 2020). Another crucial challenge for Seychelles as a SIDS with limited human resource capacity is the high turnover of members in these meetings, which limits knowledge management and coordination. Finally, these mechanisms are not meeting regularly, partly because of a lack of funding and planned meetings for each calendar year (UNDRR 2020).

## Conclusion

This article aimed to identify the challenges in integrating DRR and CCA with proposed improvement strategies. The key findings in this study confirmed that the challenges in linking DRR and CCA in Seychelles are grouped into eight categories that address issues linked to strategic, conceptual, operational, institutional and financial coherence. To further compound the problem, structural coherence was weak on the strategic rather than conceptual levels, leading to poor institutional, operational and financial coordination. The consequences, therefore, gave rise to incidental integration and collaboration on an ad hoc basis. These findings from this case study from Seychelles remain valid and lessons learned are relevant to other African countries and SIDS within the Indian Ocean Region that share similar circumstances with the Seychelles. We argue that this case study sheds light on the existing efforts and challenges for integrating DRR and CCA and proposes strategies for improvement that cut across the four priority areas of the Sendai Framework for DRR. The study will serve as a guide for Seychelles and other countries on how to effectively link DRR and CCA to minimise the duplication of efforts and enhance the efficient use of human and financial resources while concomitantly achieving the objectives of DRR – to reduce vulnerability and enhance resilience.

## Appendix 1

**TABLE 1-A1:** Summary of stakeholders interviewed by institutions and organisations.

Name of institution or organisation	No. of participants	Type of organisation
DRMD	6	Government
SMA	2	Government
MACCE	8	Government
Land Transport Department	2	Government
Tourism Department	2	Government
Ministry of Local Government and Community Affairs	3	Government
SeyCCAT	3	Public-Private Trust
University and Research Institutes	4	Academia
S4S	1	NGO
TRASS	1	NGO
Local environmental consultants	6	Private sector
UNDP	2	International NGO

DRMD, Disaster Risk Management Division; SMA, Seychelles Meteorological Authority; MACCE, Ministry of Agriculture, Climate Change and Environment; SeyCCAT, Seychelles Conservation and Climate; S4S, Sustainability for Seychelles; TRASS, Terrestrial Restoration Action Society; UNDP, United Nations Development Programme; NGO, Non-Governmental Organization.
